# Do Children Need Adult Support During Sociodramatic Play to Develop Executive Functions? Experimental Evidence

**DOI:** 10.3389/fpsyg.2021.779023

**Published:** 2021-12-06

**Authors:** Nikolai Veresov, Aleksander Veraksa, Margarita Gavrilova, Vera Sukhikh

**Affiliations:** ^1^School of Education Culture & Society, Monash University, Melbourne, VIC, Australia; ^2^Faculty of Psychology, Lomonosov Moscow State University, Moscow, Russia; ^3^Laboratory of Psychology of Childhood and Digital Socialization, Psychological Institute of the Russian Academy of Education, Moscow, Russia

**Keywords:** sociodramatic play, Child-led play, Adult-led play, Free play, executive functions

## Abstract

The cultural-historical approach provides the deep theoretical grounds for the analysis of children’s play. Vygotsky suggested three critical features of play: switching to an imaginary situation, taking on a play role, and acting according to a set of rules defined by the role. Collaboration, finding ideas and materials for creating an imaginary situation, defining play roles, and planning the plot are complex tasks for children. However, the question is, do children need educator’s support during the play to develop their executive functions, and to what extent? This experimental study was aimed at answering this inquiry. The four modes of sociodramatic play were created which differed in the adult intervention, from non-involvement in the play to its entire organization. The play could be child-led (with adult help), adult-led, or free (without any adult intervention); and there was also a control group where the children heard the same stimulus stories as the other groups but then followed them up with a drawing activity instead of a play activity. The study revealed that, firstly, the ways of educator’s involvement in the play differed in their potential in respect to the development of executive functions, and, secondly, this influence was not equal for different components of executive functions. Free play in the experiment was not a beneficial condition for the development of any of the studied components of executive functions, compared to the conditions involving the participation of an adult in the play. Furthermore, the type of adult intervention stimulated the development of various executive functions. The entire organization of the play by the adult had a positive impact of their general development. In contrast, the adult’s assistance in the organization of the children’s play had a positive effect on the development of inhibitory control. The study results can be helpful when considering educational practices within a cultural-historical approach to engaging the potential of play in children’s learning and development around the world.

## Introduction

Play is one of the most vigorous activities in human life (Lillard, 2017; Fleer and Hammer, 2019; Nykiforuk et al., 2019). In addition to enjoyment and a sense of community, it allows participants and observers to empathize with, experience, and make sense of real or imagined situations (Lillard, 2007; Miller et al., 2009; Vasc and Lillard, 2020; Veraksa et al., 2020). Nevertheless, the phenomenon of play is not limited to this (Veraksa and Sukhikh, 2020). It can also be seen as a form of recreation and a way of releasing tension or, on the contrary, as a mean for training certain necessary skills (Pirrone and Di Nuovo, 2014) and decision-making abilities (Bodrova and Leong, 2017; Foley, 2017). A common basis for all manifestations of play is that they are devoid of coercion, and their visible and semantic fields do not coincide (van Oers, 2013; Veraksa and Sukhikh, 2020). This mismatch can be expressed in several ways: for example, a child who pretends to be a doctor realizes that he/she is not a doctor but acts in accordance with this role (Vygotsky, 2016). Above mentioned field mismatch can also be observed in the child’s use of one object that actually represents another (substitute object) (Barnett et al., 2006; Vieillevoye and Nader-Grosbois, 2008). For example, a child might use a pencil instead of a syringe to give a shot while playing.

The divergence of visible and semantic fields becomes possible in preschool age through the development of imagination (Nicolopoulou, 2010; Fleer, 2011; Li et al., 2015; Lillard and Taggart, 2019). Due to his/her imagination, the child is exposed to acting not directly but symbolically through play activities (Kelly et al., 2011; Carlson et al., 2014; Delvecchio et al., 2016). In play activities, using objects or imagery, the child replicates adult actions (cooking, taking care of children, talking on the phone) or plays out examples of the relationships of others (e.g., meeting dolls, playing together) (Nicolopoulou and Ilgaz, 2013; Pierucci et al., 2014; Harris, 2016; Orekhova et al., 2020). According to researchers who have developed an understanding of play within the cultural-historical approach, in such symbolic, playful actions children model attitudes and understanding of the adult world and become aware of their desires (Elkonin, 1980, 2005; Vygotsky, 2004; Smirnova, 2015, 2017). At the same time, despite the comprehension of the difference between reality and play experience (pretending), the child gets satisfaction from a symbolic play of the story (Pierucci et al., 2014).

Children’s play can vary significantly depending on age, culture, subject environment, living conditions, and other factors (Trawick-Smith et al., 2015; Li et al., 2016; Børve and Børve, 2017). Nevertheless, various approaches have highlighted types of children’s play and their common sustainable components (e.g., review Veraksa et al., 2020). Within the cultural-historical approach, the play has received particular attention given its consideration as a leading activity in preschool age (Veresov, 2006; Edwards, 2014; Veresov and Barrs, 2016). In other words, play in the cultural-historical approach is understood as an activity in which changes in the mental development of children become possible (Veresov, 2006). Not only have types of play been allocated, but the periodization of its development in preschool-age has been developed within the framework of the cultural-historical approach (Veresov and Barrs, 2016). According to the periodization proposed by Elkonin, from 2 to 3 years, children go through the first stage of play development – *object-manipulative play*. The child plays with objects representing objects of real-life (cars, dolls, dishes) in which represents simple actions of adults (pouring tea, rolling cars). At this stage, the child does not need play partners. From the age of three to four, the child passes the second stage – *role play*, in which the child represents people and animals (doctor, tiger cub, kitty). At this stage, the child still does not need play partners, and the role rules are not formulated, neither tracked. In the transition to the third stage of *sociodramatic play* between the ages of five and seven, the child tends to play with other children. Roles and rules are outlined even before the play begins, and they determine and direct the plot and behaviour of children.

According to the cultural-historical approach, sociodramatic play contains the most significant number of opportunities to develop executive functions at preschool age. This type of play has three key characteristics: children create an imaginary situation, assume roles and follow a set of rules dictated by the roles (Vygotsky, 2004; Vasc and Lillard, 2020). For example, children may play in a hospital (imaginary situation) where roles are assigned before the play begins. The role consists of performing duties and exercising corresponding rights (Vasc and Lillard, 2020). For example, the doctor should invite the patient into his/her office, listen to the complaint and offer a treatment; and the patient should provide information and follow the doctor’s recommendations. At the same time, children control the performance quality and demand that their playmates respect the characteristics and the boundaries of their roles. Studies have demonstrated that different children can develop sociodramatic play differently (Veraksa and Sukhikh, 2020). A high level of sociodramatic play involves (a) the child’s unrestricted ability to find substitute objects and use them as means for the reproduction of a certain situation in the play; (b) assuming the role to play out the situation; (c) the ability to construct, develop and meaningfully enrich play plots; (d) the ability to dialogue with the coplayers regarding the roles distribution and plot planning; and (e) mastery of the skills of cooperation and collaboration with peers to continue the play episode for several hours (Elkonin, 1999/2005).

### Adult Support During Sociodramatic Play

Sociodramatic play is a multidimensional culturally determined form of activity (Elias and Berk, 2002). In sociodramatic play children depict situations from real life, that is, they master cultural content (Shuttleworth, 2011; Whitebread, 2012; Karabon, 2017). Traditionally, because adults are the bearers of cultural content and ideal forms, it is believed that they should facilitate the child’s learning and development (Fleer et al., 2017). This probably also applies to play as one of the main activities of preschool children. A truly rich play is possible if a child is not only familiar with the diversity of reality around but also has an idea of a wide range of roles, can create an imaginary situation, arrange the space for the play, create a plot, use substitute objects, select materials for creating costumes and play attributes (Smirnova, 2015; Ryabkova et al., 2019). It can be assumed that this explains the fact that plays range from short and simple (daughters-mothers, school, store) to complex and lengthy (launching a spaceship, travelling to another country) (Smirnova, 2017).

The listed play “skills” are in the zone of proximal development of preschool children and therefore, require adult involvement (Verenikina, 2003). However, there is still no clear understanding of optimal strategies of this intervention into a sociodramatic play (Whitebread and O’Sullivan, 2012). Today, with the understanding of the developmental potential of play and the spread of play technologies such as playworlds (Fleer et al., 2020) in preschool education, conducting such research is of particular importance (Veresov and Barrs, 2016; Loizou, 2017; Samuelsson, 2020). Relevant questions are, for example, to what extent should the adult be involved in the play? What role should he or she preferably take in its framework: an observer, a facilitator, a participant, or an organizer? How to support children’s initiative while playing and not to impose ready-made plots on them? Answers to these questions will help find the necessary balance in accompanying adult sociodramatic play and not reduce it to a dramatization or theatrical productions in which children almost completely lose subjectivity (McCabe, 2017).

### Play and Executive Functions

Executive functions are a group of cognitive skills that support voluntary behaviour and the process of purposeful problem solving (Garon et al., 2008; Friedman and Miyake, 2017; Morosanova, 2021). Core components of executive functions (working memory, cognitive flexibility, and inhibitory control) are associated with children’s academic and personal achievements, as demonstrated by numerous cross-sectional and longitudinal studies (Garon et al., 2008; Poon, 2018; Houwen et al., 2019). Executive functions actively develop during preschool age and also lend themselves to some degree of purposeful shaping (Chavez-Arana et al., 2018). Research has shown that specifically designed adult sports, dance, and training activities have meaningful positive effects on the development of working memory, cognitive flexibility, and inhibitory control (Diamond et al., 2007; Diamond and Ling, 2016). The size and duration of the effect varies and depends on a variety of variables (length and form of sessions, group size, and children’s engagement). Nevertheless, the possibility of purposeful development of executive functions is an ascertained fact, and it entails the need to find the best and the most effective means to achieve this goal.

All of these are grounds to believe that play can be considered as a space for training of children’s executive functions in preschool age (Shaheen, 2014; Fleer et al., 2017; Veraksa et al., 2019, 2020). First, play is built on submission of the child’s behaviour to rules, roles and a plot. The child’s full participation in the play activity becomes possible when the essential components of executive functions are involved (Bukhalenkova et al., 2021). For example, working memory is required to retain the story, the set of role rules, and focus on the meaning of substitute objects and the play space (Barnett et al., 2017). Inhibitory control ensures that impulsive actions are restrained in favour of voluntary and appropriate actions for the role being played. Cognitive flexibility is necessary to switch between real and imagined situations. Second, the play relies on the child’s motive orientation (Hedegaard, 2012; Veraksa and Sukhikh, 2020) and engages the child by itself (Whitebread, 2017; Bondi and Bondi, 2021). It was proved that due to playing motivation (natural desire to play); children handle their behaviour better than under the conditions of laboratory experiments or specially organized activities (Istomina, 1975).

Kravtsov and Kravtsova described a study on school readiness of 5–6-year-old children conducted in the 1970s under the guidance of Elkonin (Kravtsov and Kravtsova, 2013). It was assumed that school readiness was related to the motivation and ability of performing some monotonous actions at the request of a teacher. In order to assess the ability to subordinate their behaviour to such an external task, in the laboratory, children were asked to move small objects (matches) from one place to another one by one. The experimenter, having assigned the task, left the room and quietly observed their behaviour. At first, most children obediently focused on the task and were moving the matches one by one. Then they started to take two or three matches or whole handfuls of them, or began to switch to completely different activities (examining the room, playing with the objects they could find there, etc.). One child, who actually spent a long-time moving match one by one, was an exception. When the child was done with his activity, the experimenter inquired about how the task went. The child shared that he played as if he was a crane and needed to carry bricks to build a house. This case indicates that in a play situation, children can maintain their interest in the task longer.

Also, apart from play motivation, it is worth mentioning the communication motivation, which is also inherent in play (Evaldsson and Tellgren, 2009; Pálmadóttir and Johansson, 2015). If a child is inattentive, does not remember the conditions of the play activity or regularly disobeys the rules, peers will be less willing to agree to play with him/her. The need for communication also encourages the child to behave purposefully and remember the actions and plot being played out (Mathieson and Banerjee, 2011). Research confirms that children with developed skills in organizing and participating in cooperative play are indeed more attractive to peers than non-involved children (Veraksa and Bukhalenkova, 2020). Thirdly, play is a unique space for the realization of the child’s initiative: there, the child gets the experience of realization of his/her ideas and goals earlier and easier (Whitebread, 2017; Bondi and Bondi, 2021). The limitless possibilities of the imaginary situation allow the child to recreate and live out almost any situation in the play. Early experimental work has already documented the developmental potential of play for the formation of executive functions (Blankson et al., 2012; Bodrova et al., 2013; Veraksa et al., 2019, 2020; Doebel, 2020; Veraksa and Bukhalenkova, 2020; Vidal Carulla et al., 2021). However, the mode of adult intervention in play has not been analyzed as a factor that may influence children’s unique play experience and, therefore, condition the developmental process of executive functions.

### Current Study

This experimental study was aimed at elucidating the influence of different types of teacher’s participation in a sociodramatic play on the development of executive functions in children. There were three variants of experimental conditions, and all of them were based on the same play plots, with an identical set of play attributes and roles. However, they differed in the adult intervention, and how play sessions were organized: from the non-participation of the adult in the play (Free play) to its entire organization (Adult-led play). The intermediate condition implied the adult helping one of the children take the position of the leader in the play (Child-led play). The study’s overall goal was to clarify the extent to which the adult should be involved in a sociodramatic play to create the most favorable conditions for the development of executive functions. The following research questions were framed: (1) are there any significant differences in the development of children’s executive functions depending on the type of adult intervention into their sociodramatic play; and (2) which way of adult intervention in a sociodramatic play is most effective in terms of children’s development of executive functions? The study assessed all the main components of executive functions using five tests to answer these questions fully.

Regarding the first research question, we assumed that different modes of adult intervention in sociodramatic play would have different effects on the development of executive functions in children. In other words, it was hypothesized that the type of adult intervention had a meaningful effect on the course of children’s sociodramatic play and determined the child’s unique experience in a great measure. Regarding the second research question, we hypothesized that the most significant progress in the development of executive functions would be demonstrated by participants under the Child-led play experimental condition. The hypothesis is justified by the fact that each child in this condition has the opportunity to take the leading role with the help of an adult. Such an experience engages working memory (the child controls the development of the plot, and must remember the roles of all the participants), inhibitory control (the child assumes the responsible role of director, regulates the behaviour of other children), and cognitive flexibility (the child controls the overall plot and actions of the play characters). It was also assumed that the Adult-led play condition would have a positive effect on inhibitory control and cognitive flexibility, as the adult reverted to the role when necessary and helped children to orient themselves in the play context. It was suggested, however, that the entire adult organization of the play reduced its stimulating potential for the development of working memory, since the development of the plot and the distribution of roles was controlled by the adult. Based on the theoretical model of the cultural-historical approach to the understanding of play, Free play has the least developing effect, because the adult does not interfere in the course of the play and does not help in the construction and development of the plot. Under such conditions, the play will probably collapse because of difficulties related to independent planning and organization of the play by children.

## Materials and Methods

### Participants

A total of 136 typically developing children (51.8% boys) 5–6 years old (*M* = 60.79; SD = 4.10 months) participated in the study. All children were attending public kindergartens in the districts characterized by the same infrastructure level and designed to accommodate primarily medium-income families. Since children in Russia are assigned to the kindergartens according to their registered residence address. This allows us to infer that the sample is homogeneous in terms of the family’s socioeconomic status. Five techniques were used in the pre-test and post-test to assess the main components of executive functions. The assessment of executive functions in children was carried out twice individually by trained research assistants. The pre-test was conducted during a week before the experiment, and then a post-test was done during a week after the end of the experiment. The Ethics Committee approved the study and consent procedures of the Faculty of Psychology at Lomonosov Moscow State University (the approval No: 2021/72). All parents provided written informed consent for their child to take part in the study.

### Materials

*Inhibition* subtest (Korkman et al., 2007) was used to measure children’s ability to inhibit automatic cognitive responses. It included two series of shapes (circles/squares, and arrows). In the first part, the child was asked to name the shape or the direction of the arrow(s) (naming trials). In the second part of the task, the child was asked to name the shape or the direction conversely: to name circles when squares were presented and vice versa (inhibition trials).

*Dimensional Change Card Sort* (DCCS, Zelazo, 2006) was used to measure cognitive flexibility. In this task, children were asked to sort, in three rounds, cards depicting colored objects, according to different rules. The first sorting was based on the picture’s color (pre-switch trials), the second, on the object’s shape (switch trials), and the third sorting included a conflicting rule: the sorting principle (color or shape) was indicated by the presence or absence of a frame on the card (post-switch trials).

*Statue* subtest (Korkman et al., 2007) was used to assess motor persistence. It requires a child to silently maintain a static body position with the eyes closed for 75-s. The child is instructed to not respond to sound distracters, which the experimenter makes 4 times. Four scores were computed for the Statue subtest – Statue, Body Movement, Eye Opening and Vocalization, and a Total score. The tester recorded the number of movements the child made in 5-s intervals (e.g., head turning, eyes opening or vocalizing and/or laughing).

*Memory for Designs* subtest (Korkman et al., 2007) was used to assess visual working memory. Two parameters of visual memory were measured – memorization of pictures (selection of pictures, as in a presented sample, from an array of similar pictures) and memorization of pictures’ spatial arrangement (recall the cards’ position in a sample).

*Sentence Repetition* subtest (Korkman et al., 2007) was used to assess verbal working memory. The test contained 17 sentences, gradually increasing in their complexity (in terms of length and syntactical structure).

### Procedure

For 7 weeks, two play sessions a week were conducted with the children in each experimental series. Overall, the children attended 14 play sessions in each series, which amounted to 4–4.5 h of experimental exposure. The children were divided into groups of five for the interventions. For play meetings, children were offered sets of roles setting a specific play context (common for all three experimental conditions) and the same materials for creating costumes and play attributes. Plots, roles and play attributes were planned according to the principle of agenderism, thus allowing each child, regardless of sex, to take any role in the play. In Child-led play intervention, the adult helped the child to take a “director’s position” – that is, to distribute roles, think up a plot and act it out with other children. Two scenes were acted out in one play session with two children as directors. Thus, each of the children had a chance to lead a play an average of four times during the experiment. In Adult-led play intervention, the distribution of roles and the acting out the story was supervised by an adult: the experimenter told the story, and children acted according to the plot and their roles. In Free play intervention the adult helped the children only at the initial stage: offered them a plot, and provided role attributes, but did not interfere anymore during the course of the play. In the control group, meetings were conducted with the same frequency and duration as under other conditions. However, the experimenter only read to children the stories played out under the Child-led play and Adult-led play conditions. Control group participants listened to the stories and made drawings based on them. Thus, under all conditions, the children spent an equal amount of time in the class and were exposed to identical stories. The only difference consisted of how children acted out the offered story: independently, with the help of an adult or required the entire play to be organized by an adult.

Under all conditions, the experimenters followed the protocol specified for the play sessions: (1) establishment of contact with children; (2) introduction and rules setting: “We are going to play today. Each of you will be assigned a role, and you will act it out”; (3) arranging the space in accordance with the play context (a kingdom, a jungle, or the space) by means of modular construction blocks, furniture pieces, and fabric (the exact technology depended on the experimental conditions); (4) role “immersion” – the leader explains the main features of the characters (for example, “the evil wizard is very powerful, he knows all magical secrets”), and suggests to the participants playing their characters using mimics, gestures, words, and sounds; (5) creation of costumes – the leader invites children to the place where all the attributes are kept, so that they could choose the necessary ones and make their costumes (the experimenter and his/her assistants help the participants); (6) beginning of the play – the leader invites everyone to immerse into the play context, for example: And now, let’s imagine, we are in… (a kingdom, a jungle, or the space); (7) the course of the play – the assistant only intervenes if discipline maintenance or prevention of unacceptable behaviour is required; and (8) end of the play – the experimenter warns the children that the time is running up, and then stops the play. Afterward, the experimenter and his/her assistants help the children to take off their costumes and ask them to share their impressions.

### Statistical Analyses

One-Way ANOVA (Welch’s) was used to compare pre-test children’s performance in executive function tasks age for each of the four experimental play conditions. For the main analysis One-way repeated measures ANOVA with four levels of the within-subjects factor “time” (pre-test and post-test), and four levels of the between-subjects factor “play condition” (Child-led play, Adult-led play, Free play, and Control group) was used to determine main and interaction effects of Play condition and Time for each of the executive functioning measures. Level of significance was set at *p* < 0.05 for all analyses. Partial eta square (partial η^2^) was reported as an estimation of effect size. According to the rules given by Cohen (1988) for eta-squared effect size interprets as follows: η^2^ ≤ 0.01 as small, η^2^ ≥ 0.06 as medium, and η^2^ ≥ 0.14 as large effect sizes. Power analysis showed that for a 95% probability of correctly rejecting the null hypothesis of no significant effect with the present study sample size (*n* = 136), the effect size would be ≥0.032.

## Results

Table 1 presents the descriptive statistics for pre-test study variables (executive functions measures, age) for each of the four experimental play conditions. We used One-Way ANOVA (Welch’s) for the analysis of variances between pre-test children’s performance in executive function tasks age for each of the four experimental play conditions.

**TABLE 1:** Descriptive statistics for pre- and post-test children’s performance in executive function tasks for each of the four conditions.

Executive function measure	Play condition	Pre-test				Post-test		
		*N*	*M*	SD	*p*	*M*	SD	*p*
Cognitive flexibility	Child-led play	38	17.92	1.95	0.147	19.2	2.55	0.964
	Adult-led play	28	17.71	2.59		19.1	2.67	
	Free play	34	18.18	2.66		19.1	3.10	
	Control group	26	19.12	2.36		18.9	2.70	
Inhibition control	Child-led play	37	8.22	3.27	0.086	11.9	3.26	0.156
	Adult-led play	27	9.59	2.96		11.9	4.27	
	Free play	31	8.71	3.54		10.2	3.47	
	Control group	25	9.80	1.83		11.7	3.04	
Motor persistence	Child-led play	23	26.87	2.82	0.053	27.9	2.83	0.002
	Adult-led play	23	23.83	5.33		27.3	4.27	
	Free play	28	26.21	3.33		25.4	4.70	
	Control group	22	24.59	4.33		23.0	5.18	
Visual working memory	Child-led play	27	63.63	11.97	0.222	72.1	20.04	0.764
	Adult-led play	23	63.96	13.14		71.1	18.84	
	Free play	28	63.64	18.32		73.6	19.46	
	Control group	25	72.40	18.52		76.8	19.98	
Verbal working memory	Child-led play	38	18.39	3.10	0.096	18.7	2.90	0.439
	Adult-led play	28	16.32	3.95		17.2	4.14	
	Free play	34	17.21	3.72		18.5	4.14	
	Control group	26	16.81	3.69		18.5	3.51	

*The tests to assess executive functions were performed in three separate meetings with each child, on different days, so that the time for each meeting did not exceed 15 min. For this reason, some children were not assessed for all the tests because they were not present in the kindergarten on the testing day(s).*

No significant group differences were found between experimental conditions in pre-test children’s performance in executive function tasks (*p* > 0.05). There were also no significant group differences evident in terms of age. Therefore, those variables were not included as a covariate in subsequent analyses. Still, approaching significance group differences for pre-test children’s performance were found in the motor persistence task (0.053), so pre-test was used as a covariate in the further analyses.

Next, One-way repeated measures ANOVA with four levels of the within-subjects factor “time” (pre-test and post-test) and four levels of the between-subjects factor “play condition” (Child-led play, Adult-led play, Free play, and the Control group) was performed for each of executive functioning variables. Gender was not significant predictor or part of any significant interaction effects (*p* > 0.05) so that it was dropped from the analysis.

Analyses revealed a significant main effect of Time for cognitive flexibility (*F*(1,121) = 10.81, *p* < 0.001, η^2^ = 0.027), verbal working memory (*F*(1,122) = 21.62, *p* < 0.001, η^2^ = 0.019), inhibitory control (*F*(1,116) = 2.97, *p* < 0.001, η^2^ = 0.109), visual working memory (*F*(1,99) = 20.451, *p* < 0.001, η^2^ = 0.044), and motor persistence (*F*(3,92) = 30.94, *p* < 0.001, η^2^ = 0.055). Hence, all executive measures improved significantly from pre-test to post-test. However, the effect sizes were small (for cognitive flexibility, verbal, visual working memory, and motor persistence), or medium (for inhibitory control).

**FIGURE 1:**
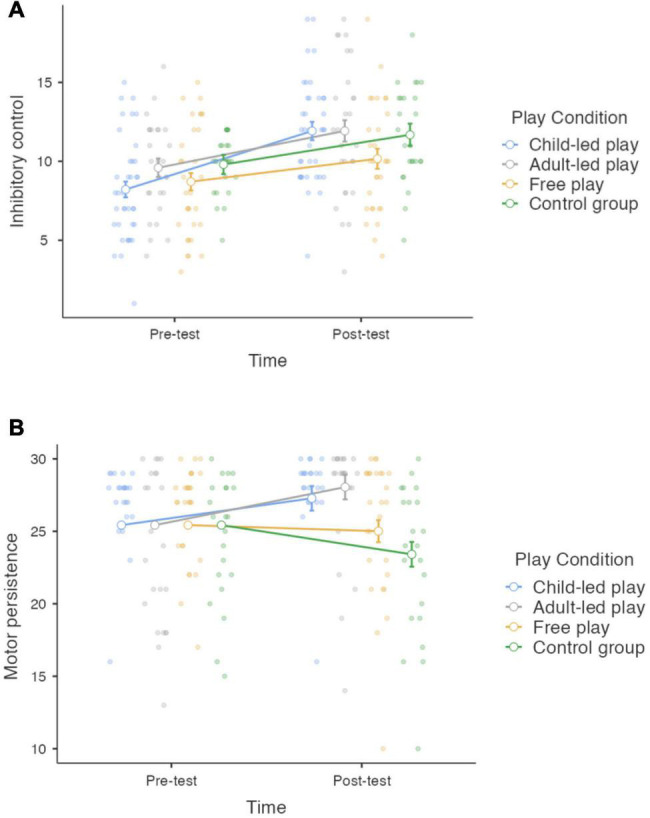
Representation of marginals means and error bars (representing confidence interval of the mean) for **(A)** inhibitory control and **(B)** motor persistence by Time and Play Condition.

The interaction effects Time × Play condition and the main effect of Play condition were of our specific interest. The Time × Play condition interaction was significant with small effect size for inhibitory control (*F*(3,116) = 2.97, *p* = 0.035, η^2^ = 0.017) (Figure 1A) and motor persistence (*F*(3,92) = 6.46, *p* < 0.001, η^2^ = 0.034) even when pre-test used as a covariate in the analyses (Figure 1B). Multiple comparisons (Bonferroni corrected) proved that children that participated in Child-led play improved more in inhibitory control than children in Adult-led play (Mean Difference = 3.71, *p* < 0.001), Free play condition (Mean Difference = 3.21, *p* = 0.003), and Control group (Mean Difference = 3.46, *p* = 0.003). As seen from Figure 1A, the largest increase in in the pre-test to post-test inhibitory control scores was noticed in children who attended the Child-led play and Adult-led play sessions. At the same time, the increase in scores for children from the Free play and Control group was less marked. When to motor persistence, multiple comparisons (Bonferroni corrected) revealed that children who participated in Adult-led play improved more than children in Control group (Mean difference = 4.64, *p* = 0.005). As seen from Figure 1B, there was a tendency to increase the pre-test to post-test motor persistence for children who had experience in a Child-led play and Adult-led play. Meanwhile, the children who were involved in the Free play condition and Control group, demonstrated a decrease in the pre-test to post-test motor persistence.

A main effect of Play condition with small effect size was registered only for motor persistence (*F*(3,92) = 6.46, *p* < 0.001, η^2^ = 0.034). Multiple comparisons (Bonferroni corrected) showed that children who had played Child-led play improved more in motor persistence than children in the Control group (Mean difference = 1.93, *p* < 0.012); and children who had played Adult-led play also improved more than children in the Control group (Mean difference = 2.32, *p* = 0.001).

## Discussion

This study was aimed to clarify, which mode of adult intervention into children’s sociodramatic play created more opportunities for the development of their executive functions. To this end, an experiment was conducted with different conditions for sociodramatic play. The results of the study are discussed below, according to the research questions posed. To the first research question, of whether there are significant differences in the development of executive functions in children depending on the adult’s intervention in sociodramatic play, according to the results obtained, the answer is positive. Indeed, throughout the experiment, children’s gains in the development of executive functions were explained by the time between pre-test and post-test and the role an adult played in the play. In Child-led play where an adult helped the participants assume a directing position (to assign roles, invent a plot, and act it out with other children), children made more noticeable progress during the experiment than under Adult-led and Free play conditions. On the other hand, in the Adult-led play, the children demonstrated greater progress in their development than in the Child-led and Control group. These findings emphasize that the type of adult intervention in a sociodramatic play has a significant effect on the course of children’s play and, to some extent, determines the child’s experience, which results in differences in the development of individual executive functions. However, not all components of the executive functions subject of this study were sensitive to variation of adult intervention. Of all the components, only the changes in inhibitory control and motor persistence could be explained to some extent by this factor. No significant effects of any play condition on individual children’s gains in cognitive flexibility and auditory working memory were registered. None of the types of play had a significant adverse effect on executive functioning development, either.

The second research question implied, which type of adult intervention in Sociodramatic play was the most effective in terms of the development of executive functions. The study revealed a slight advantage of Adult-led and Child-led play for motor persistence and inhibitory control development, respectively. Under Free play conditions, none of the studied executive functions developed more intensively than under other circumstances. Observations made during the experiment indicate that in Free play, it was difficult for children to plan and organize the process without adult assistance. For example, according to the observations of the experimenter, Free play was often interrupted because children could not agree with each other on the plot or distribute roles.

Earlier experimental works have already registered the positive potential of play in respect to the development of executive functions (Blankson et al., 2012; Bodrova et al., 2013; Veraksa et al., 2019, 2020; Doebel, 2020; Veraksa and Bukhalenkova, 2020; Vidal Carulla et al., 2021). This study complements previous findings by demonstrating the importance of adult support for the enrichment of play conditions for the development of children’s executive functions. Obtained evidence allows suggesting, first, that the way an adult participates in a play may, to some extent, shape children’s play experiences and differentially influence the development of individual executive functions. Secondly, the participation of the adult in a play in the role of organizer and helper was associated with the tremendous success of children in the development of some executive functions, compared to the Free play. These findings empirically support play-related ideas that were formulated within the cultural-historical approach (van Oers, 2013; Veresov and Barrs, 2016; Veraksa and Sukhikh, 2020). This perspective understands play as an activity through and within which children model their perceptions of the world and adult relationships. It is of unique importance for a child’s development because it is based on intrinsic motivation and brings pleasure to the player, despite its complexity. The latter stems from the inherent characteristics of play, such as the mismatch of visible and semantic fields, the subjection of the child’s actions to role rules and plot, and the need for constant development of play and coordination of play activities with peers (van Oers, 2013).

Prolonged and complex play indeed provides many opportunities for mastering cultural content and the development of executive functions. However, according to the cultural-historical approach, without the help of an adult, play, like any other activity, may not reach a high level of development for several reasons (Veresov and Barrs, 2016; Loizou, 2017; Samuelsson, 2020). For example, a child needs to see examples of developed play with an exciting and complex plot; needs to be able to practice the skills required for the planning and organizing of the play; and needs to be able to overcome difficulties that arise within its course (see Introduction). The listed skills are in the zone of proximal development of children and therefore, require adult intervention. Especially interesting in this respect, it is especially interesting that Free play in this experiment was not a winning condition for the development of any of the studied components of executive functions compared to conditions involving the participation of an adult in the play. It is likely that the participation of an adult in the play created some conditions for the practice and development of specific executive functions and increased the developmental potential of the play. Thus, the degree of involvement of the adult (organizer or helper) probably stimulates the development of different executive functions. For example, this research has demonstrated that the adult’s entire organization of the play had a positive effect on the development of executive functions. In comparison, the adult’s assistance in the organization of the play by children had a positive effect on the development of inhibitory control. In both cases, the adult took on the task of planning the play (preparation of the script, help in definition of the roles). Probably, play including a plot and specific roles creates more opportunities for training those executive functions in preschool children. The specific influence of Child-led play on the development of executive functions could also be explained by the fact that the child undertakes planning, organizing, role assignment, developing the play-plot, and monitoring peers playing actions. However, these results need to be taken with caution, due to the medium effect size. More research is needed to replicate this result and, hence, the reliability of the effect.

### Limitations

The present findings must be interpreted in light of a few limitations. The pre-test and post-test were conducted within 1 week before and after the experiment due to the organizational and technical capabilities of the research team. Thus, the post-test is not immediate after the end of the intervention. Finally, limited statistical power because of the modest sample size in the present study (*N* = 136) may have played a role in limiting the significance of some of the statistical comparisons conducted. A power analysis revealed that for a 95% probability of correctly rejecting the null hypothesis of no significant effect with the present study sample size (*n* = 136), the effect size would be ≥0.032. Hence results would have been more promising with a larger sample size. Given the power analysis outputs and the present study sample size, it is important to be cautious about the results obtained, especially those with small effect sizes.

## Conclusion

In light of the increasing interest in play pedagogy worldwide, one of the greatest challenges is identifying the extent and the form of optimal adult involvement in children’s play. The cultural-historical approach provides the deep theoretical grounds for play experience analysis. This experimental study attempted to evaluate the effect of different types of educator’s support during the play on the development of executive functions in preschool children. Several experimental conditions were created in the study, which differed in the role of an adult: from non-participation to the entire organization of the play. The findings indicate that Free play has no effect on the development of executive functions; while Child-led play has the medium size impact on inhibitory control and motor persistence and there is also a slight positive impact on these executive functions in Adult-led play. The study outcome can be helpful when considering educational practices within a cultural-historical approach to engaging the potential of play in children’s learning and development around the world.

## Data Availability Statement

The raw data supporting the conclusions of this article will be made available by the authors, without undue reservation.

## Ethics Statement

The studies involving human participants were reviewed and approved by the Ethics Committee and consent procedures of the Faculty of Psychology at Lomonosov Moscow State University (the approval No: 2021/72). Written informed consent to participate in this study was provided by the participants’ legal guardian/next of kin.

## Author Contributions

NV, AV, MG, and VS contributed to conception and design of the study and wrote sections of the manuscript. VS organized the experiment and database. MG performed the statistical analysis. NV and AV wrote the first draft of the manuscript. All authors contributed to manuscript revision, read, and approved the submitted version.

## Conflict of Interest

The authors declare that the research was conducted in the absence of any commercial or financial relationships that could be construed as a potential conflict of interest.

## Publisher’s Note

All claims expressed in this article are solely those of the authors and do not necessarily represent those of their affiliated organizations, or those of the publisher, the editors and the reviewers. Any product that may be evaluated in this article, or claim that may be made by its manufacturer, is not guaranteed or endorsed by the publisher.
